# Does Daily Intake of Matcha Tea Enhance The Periodontal Health of Patients With Localised Gingivitis: A Control Study

**DOI:** 10.3290/j.ohpd.c_2268

**Published:** 2025-09-11

**Authors:** Rasha Salah Abood, Firas Bashir Hashim Al-Taweel, Hadeel Mazin Akram, Hayder Raad Abdulbaqi

**Affiliations:** a Rasha Salah Abood Researcher, MSc. in Periodontics, College of Dentistry, University of Baghdad, Iraq. Study design, conducting and collating the research, writing the manuscript.; b Firas Bashir Hashim Al-Taweel Researcher, PhD. in Periodontics, College of Dentistry, University of Baghdad, Iraq. Study design, conducting and collating the research, writing the manuscript.; c Hadeel Mazin Akram Researcher, MSc. in Periodontics, College of Dentistry, University of Baghdad, Iraq. Study design, conducting and collating the research, writing the manuscript.; d Hayder Raad Abdulbaqi Researcher, PhD. in Periodontics, College of Dentistry, University of Baghdad, Iraq. Study design, conducting and collating the research, writing the manuscript.

**Keywords:** gingivitis, matcha tea, 8-OHdG, saliva

## Abstract

**Background:**

Gingivitis is a reversible, dental biofilm–induced inflammation which is characterised by bleeding on probing (BOP) and oxidative stress in the gingival tissues. Matcha tea, a type of green tea with high antioxidant content, has been reported to enhance periodontal health, but its exact beneficial efficacy remains to be investigated.

**Objective:**

This pilot-controlled trial aimed to investigate the influence of daily consumption of matcha tea on gingival status and salivary 8-hydroxy-deoxyguanosine (8-OHdG) concentration in localised gingivitis patients, as well as to determine the bleeding on probing (BOP) threshold that predicts for gingival health after intervention.

**Methods:**

Twenty-seven adults with localised gingivitis (BOP >10% but ≤30%) drank matcha tea twice a day for 30 days while maintaining oral hygiene habits. Clinical parameters, including plaque index (PI) and BOP, as well as salivary 8-OHdG, were evaluated at baseline and post-intervention. After treatment, patients with BO <10% were reassigned as healthy, and those with BOP ≥10% as gingivitis. Regression and correlation analyses, including the Wilcoxon and Mann–Whitney test, ANCOVA, and receiver operating characteristics (ROC) curve, were performed to determine a BOP cut-off value that would predict gingival health.

**Results:**

PI, BOP, and 8-OHdG were statistically significantly decreased in the entire cohort after 1 month. Patients reassigned as healthy underwent a greater reduction in PI (from 28.3 ± 7.5 to 17.5 ± 5.3), BOP (from 16.4 ± 5.0 to 5.6 ± 2.5) and 8-OHdG (from 6.9 ± 1.6 to 5.6 ± 1.8 ng/ml). In contrast, patients with gingivitis had statistically significant reductions in PI and BOP, but not in 8-OHdG. The mean BOP reduction was statistically significantly higher in healthy subjects. A ROC calculation revealed a BOP cut-off <18% at baseline, indicating a high likelihood of achieving gingival health following ingestion of the matcha tea (sensitivity = 0.92, specificity = 0.70).

**Conclusion:**

Daily intake of matcha tea for 1 month improved gingival health and decreased oxidative stress markers in the localised gingivitis patients. A <18% baseline BOP predicted successful transition to gingival health after intervention. These results corroborate the adjuvant therapy of matcha tea in the treatment of gingivitis, and further large-scale randomised trials are recommended.

**Source of funding:**

No external funding was received.

Periodontal inflammations are common oral health diseases with accelerated rates of incidence and prevalence.^32^ Gingivitis is the inflammation confined to the gingival tissues surrounding teeth. Individuals might develop gingivitis at any age, from childhood to old age. In literature, a high prevalence of gingivitis (approximately 50–100%) has been reported in dentate adults.^1,29^ Untreated gingivitis increases the risk for developing more advanced periodontal lesions (periodontitis) in which permanent destruction of tooth-supporting bone occurs, leading to tooth loss.^17^ Therefore, management of gingivitis is of pivotal importance for periodontal health and saving teeth.^18^

The main cause of gingivitis is the presence of dental biofilms on tooth surfaces. The persistence of this microbial deposit initiates inflammation in the adjacent gingival tissue.^6^ During this process, leukocytes, mainly neutrophils, are recruited from adjacent blood vessels to the sites of inflammation within the affected tissue.^16^ These neutrophils represent the first responder cells that counteract microbial invaders through various biological activities. For instance, neutrophils release reactive oxygen species, which participate in killing the microbial invaders^5^ in an attempt to preserve the health of the gingival tissue. However, the persistent presence of microbial deposits on the tooth surface leads to continuous recruitment of neutrophils and overproduction of reactive oxygen species. This leads to oxidative stress within the tissue; a state develops when the level of oxidants is higher than the host’s neutralising ability by antioxidants. The presence of oxidative stress in tissues exerts cytotoxic effects on host cells, leading to tissue degeneration.^27^ The oxidative stress in the gingival tissue could be measured by several biomarkers within saliva. One of them is 8-hydroxy-deoxyguanosine (8-OHdG), which is a fragment of the damaged DNA induced by oxidative stress. Such a biomarker level in saliva has the potential to reflect the activity of periodontal diseases. Elevated levels of 8-OHdG in saliva suggest a decline in the host’s ability to neutralise generated reactive oxygen species, indicating oxidative stress.^4,8,14,28^ Many studies have indicated a positive association between the salivary level of 8-OHdG and bleeding gingiva in patients having periodontal inflammation. In response to periodontal treatment, significant reductions in the salivary levels of 8-OHdG have been documented.

Green tea is a popular beverage worldwide. There is growing evidence that suggests green tea has benefits regarding periodontal health.^2,9,11,19^ However, the effectiveness of green tea as an adjunct in periodontal therapy is still under debate despite its positive impact in reducing gingival inflammation and bleeding tendency. Such uncertainty is due to the heterogeneity of studies in the literature, encouraging researchers to conduct more studies with a low risk of bias.^23^

Matcha tea is a type of green tea known for its unique flavour. It is prepared from the leaves of the green tea plant (*Camellia sinensis* L.) grown under shade for several weeks prior to harvesting. Due to these distinct cultivation circumstances, matcha tea gains its particular proportions of bioactive constituents known for their beneficial health properties, encouraging its intake worldwide. Matcha tea is rich in fibres, proteins and caffeine as well as phenolic compounds, providing its profound antioxidant properties.^15^ There is an increasing body of evidence that indicates that matcha tea has beneficial effects in enhancing gingival health. Rinsing with matcha tea reduces the salivary levels of *Porphyromonas gingivalis*, *Aggregatibacter actinomycetemcomitans*, and *Prevotella intermedia*, suggesting a beneficial effect of matcha tea in promoting gingival health.^24^ Moreover, it has been reported that consuming matcha tea on a regular basis reduces bleeding of the gingiva and plaque accumulation on teeth.^2^ Furthermore, daily intake of matcha tea has a positive impact on the oral health-related quality of life.^2^ From all the above, this study aimed to evaluate the effect of matcha tea on gingival health and the salivary level of 8-OHdG after 1 month of daily intake. Also, this study estimated the bleeding on probing (BOP) threshold that predicts for gingival health after matcha tea intake.

## METHODS

### Study Population and Methodology

#### Design

This was a pilot control trial conducted from January 2023 to February 2024. The protocol of this study was reviewed and approved by a relevant ethical committee (reference no. 419621; 27 December 2021; College of Dentistry/University of Baghdad). The participants were selected from the pool of patients attending the clinics in the College of Dentistry/University of Baghdad.

All patients agreed to voluntarily participate in this study and signed consent forms collected by the examiner. The protocol of this study was registered in clinicaltrials.gov (NCT05681325).

#### Study sample

No sample size was estimated as this study was a pilot trial involving 27 patients. The inclusion criteria included those patients who regularly brushed their teeth, diagnosed with localised gingivitis, having > 10% of sites but no more than 30% with BOP,^6^ having at least 20 teeth on intact periodontium and having no clinical attachment loss (CAL) at > 2 interdental sites or facial and/or oral CAL. Any patient who was currently pregnant or lactating, a smoker, having an orthodontic appliance, taking vitamin C supplements or having a recent history of matcha or green tea intake was excluded.

After selecting the patients, saliva samples were collected as previously reported^33^ at the baseline visit (T0). In brief, patients first rinsed their mouths with water for half a minute and then were asked to expectorate into sterile 5 ml tubes until they had filled 3 ml of unstimulated saliva. The saliva samples were then centrifuged (20 min at 3000 rpm) and stored at –20°C until further analysis. The salivary level of 8-OHdG was estimated by enzyme-linked immunosorbent assay (ELISA) according to the manufacturer’s instructions (Shanghai Yehua Biological Technology, China).

After collecting saliva samples, plaque index (PI)^25^ was recorded at four sites/tooth with the aid of a disclosing agent (EMS®, Switzerland). Then, BOP was recorded at six sites/tooth using a UNC-15 periodontal probe (PHOENIX®, UK). All clinical parameters were measured by a single calibrated examiner (HA). Before beginning the study, the examiner was calibrated on five volunteers for recording PI and BOP until an acceptable level of reproducibility was achieved, as indicated by a kappa value of > 0.85.

After recording the clinical parameters, the examiner asked the patients to consume matcha tea (Organic Japanese Matcha, Japan; Lot: T47I10P100) twice daily for 30 days. Additionally, the examiner instructed the patients to maintain their habitual oral hygiene measures without any modifications.

After 30 days (T1), saliva samples were collected, and clinical parameters were recorded in the same manner as at visit T0. In this visit, patients were re-diagnosed as healthy when they had BOP <10% otherwise, they were diagnosed as having gingivitis.^6^ Moreover, the examiner retrieved empty tea sachets from the patients in order to assess their compliance with drinking matcha tea. Finally, all patients received scaling and polishing (when needed) as well as oral hygiene instructions for treating gingivitis and maintaining their gingival health.

#### Statistical analysis

The study variables are described in terms of means and standard deviations for continuous variables and frequencies for categorical variables. The Shapiro–Wilk Normality test was used to check the normal distribution of the variables. The Wilcoxon test was used to compare paired variables, while the Mann–Whitney test was used to compare between the groups. Univariate analysis (ANCOVA) was used to compare the means of BOP between the two groups after matcha tea consumption. In this analysis, baseline BOP, baseline 8-OHdG 8-OHdG and PI after matcha tea intake were considered as covariates that might have an effect on the dependent variable. The receiver operating characteristic (ROC) curve was used to identify the cut-off value of BOP in patients with localised gingivitis who were predicted to have healthy gingiva after 1 month of matcha tea consumption. Also, the sensitivity, specificity and area under the curve (AUC) were estimated. All statistical analyses were performed using Prism 9 for macOS software and the Statistical Package for the Social Sciences (SPSS; version 26 for Mac, IBM, Chicago, IL, USA) software. The significance was considered when P < 0.05.

## RESULTS

**Fig 1:**
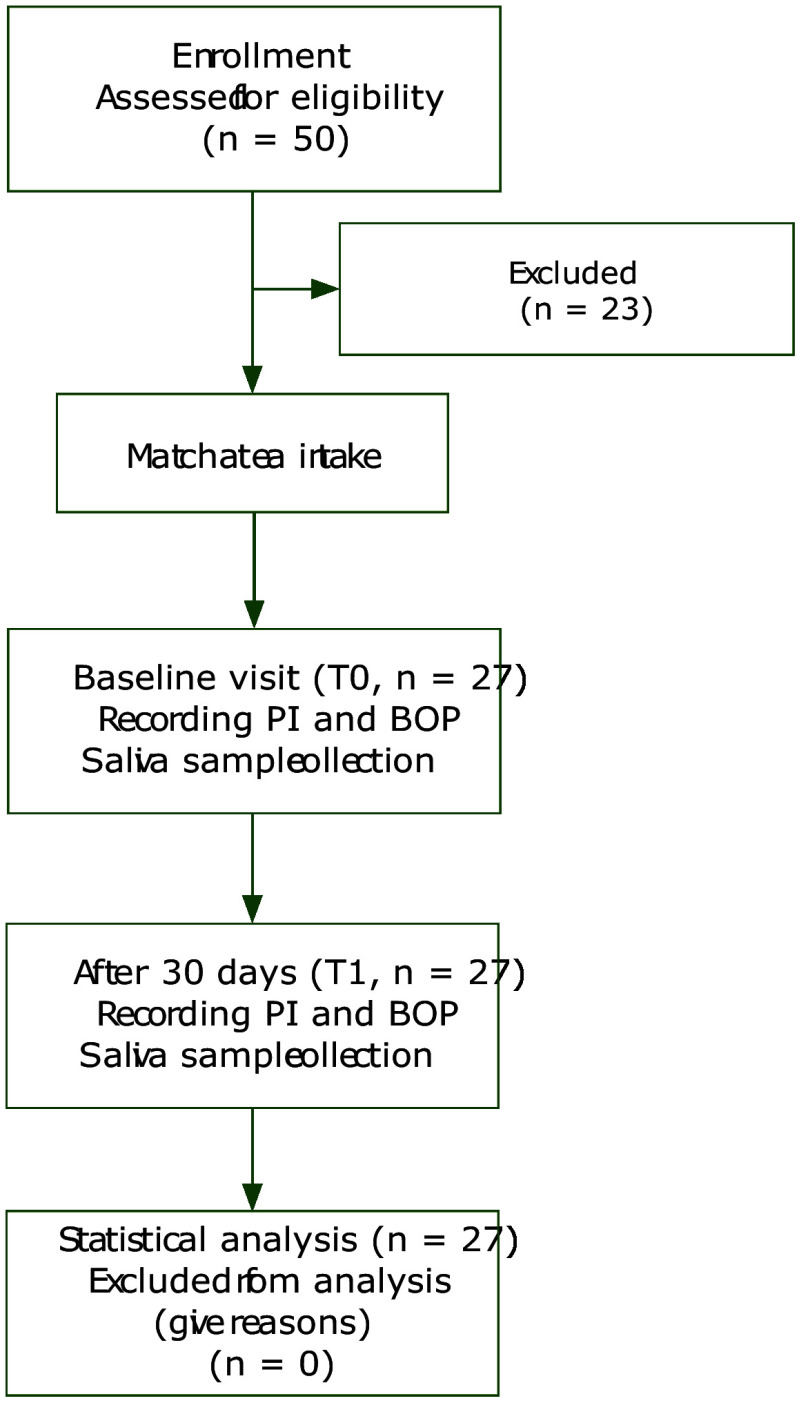
Flow of the study.

Of the 50 patients examined for eligibility, only 27 patients (26.7 ± 4.5 years) with localised gingivitis were invited to participate in this study. The majority of the patients were females (n = 25). At the T1 visit, the periodontal health of 13 patients (38 ± 4.8 years) was improved as they were diagnosed with gingival health after 1 month of matcha tea consumption. While the others (26 ± 4.4 years) were still having localised gingivitis. The flow of the study and demographic data are illustrated in Figure 1 and Table 1.

**Table 1:** Demographic characteristics of the study sample

	Total	Healthy*	Gingivitis
	Sex (n, %)
**Age (mean ±SD) years**	26.7 ± 4.5	38 ± 4.8	26 ± 4.4
**male**	2, 7.4	0, 0	2, 7.5
**female**	25, 92.6	13, 48.1	12, 44.4
*Patients in this group were diagnosed with localised gingivitis at baseline. One month after matcha tea intake, they were re-diagnosed as healthy.

**Fig 2a to c:**
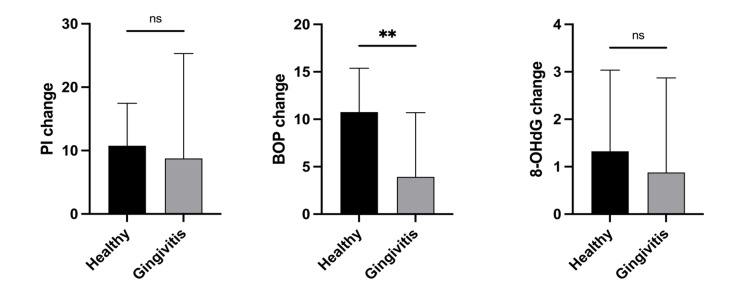
Means of change of the study variables in healthy and gingivitis-diagnosed patients after 1 month of matcha tea intake. Patients in the healthy group were diagnosed with localised gingivitis at baseline. One month after matcha tea intake, they were re-diagnosed as healthy. (a) Mean change of plaque index (PI) in healthy (10.7 ± 6.7) and gingivitis (8.7 ± 16.5) participants; (b) mean change of bleeding on probing (BOP) in healthy (10.7 ± 4.6) and gingivitis (3.9 ± 6.7) participants; (c) mean change of 8-Hydroxy-desoxyguanosine (8-OHdG) ng/ml in healthy (1.3 ± 1.7 ng/ml) and gingivitis (0.8 ± 1.9 ng/ml) participants. Comparisons by Mann–Whitney test; **significance at P value <0.001; ns: non-significant.

The means of PI, BOP and salivary 8-OHdG level were statistically significantly reduced after matcha tea intake for all patients. In healthy diagnosed patients, there were statistically significant reductions in PI (28.3 ± 7.5 to 17.5 ± 5.3), BOP (16.4 ± 5.0 to 5.6 ± 2.5) and 8-OHdG (6.9 ± 1.6 to 5.6 ± 1.8 ng/ml). In contrast, salivary 8-OHdG was not statistically different (6.1 ± 0.9 to 5.2 ± 1.9 ng/ml) in patients diagnosed with gingivitis after tea intake. Only PI (35.6 ± 15.6 to 26.8 ± 11.8) and BOP (22.0 ± 4.3 to 18.1 ± 6.7) were statistically significantly reduced (Table 2). The means of change of these variables were not statistically significantly different between healthy and gingivitis patients, except for the change in BOP, as shown in Figure 2.

**Table 2:** Means of clinical parameters and 8-Hydroxy-desoxyguanosine (8-OHdG) of the study sample

	Total (n = 27)	Healthy (n = 13)*	Gingivitis (n = 14)
T0	T1	P value	T0	T1	P value	T0	T1	P value
PI	32.1 ± 12.7	22.3 ± 10.2	<0.001	28.3 ± 7.5	17.5 ± 5.3	0.04	35.6 ± 15.6	26.8 ± 11.8	0.04
BOP	19.3 ± 5.4	12.1 ± 8.1	<0.001	16.4 ± 5.0	5.6 ± 2.5	<0.001	22.0 ± 4.3	18.1 ± 6.7	0.04
8-OHdG ng/ml	6.5 ± 1.3	5.4 ± 1.9	0.005	6.9 ± 1.6	5.6 ± 1.8	0.01	6.1 ± 0.9	5.2 ± 1.9	0.15
*Patients in this group were diagnosed with localised gingivitis at baseline. One month after matcha tea intake, they were re-diagnosed as healthy. PI: plaque index; BOP: bleeding on probing; T0: baseline visit; T1: 1 month after matcha tea intake visit. Comparisons by the Wilcoxon test; significance at P value <0.05.

**Fig 3a to c:**
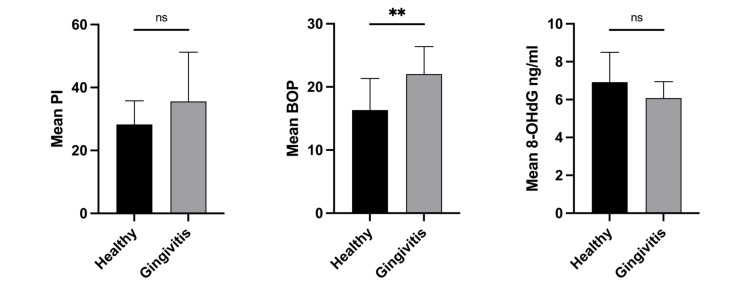
Baseline means of variables for healthy patients diagnosed with gingivitis. Patients in the healthy group were diagnosed with localised gingivitis at baseline. One month after matcha tea intake, they were re-diagnosed as healthy. (a) Mean of plaque index (PI); (b) mean of bleeding on probing (BOP); (c) mean of 8-Hydroxy-desoxyguanosine (8-OHdG). Only the means of BOP were significantly different between healthy individuals and patients diagnosed with gingivitis. Comparisons by Mann–Whitney test; **significance at P value <0.001; ns: non-significant.

At baseline, the means of PI and salivary 8-OHdG were not statistically significantly different between healthy and gingivitis patients, as shown in Figure 3. After matcha tea intake, a statistically significantly lower mean PI was detected in healthy patients, while the level of salivary 8-OHdG did not differ between healthy and gingivitis patients. On the other hand, the baseline mean of BOP was different between the two groups (Fig 3). After considering the possible effects of baseline BOP, baseline 8-OHdG, and mean PI after matcha tea intake, the mean of BOP was found to be statistically significantly different between healthy and gingivitis-diagnosed patients after matcha tea consumption; estimated marginal means were 8.1 ± 1.5 and 15.7 ± 1.4, respectively (Table 3).

**Table 3:** Univariate analysis of bleeding of probing (BOP) between healthy and gingivitis diagnosed, after 1 month of matcha tea intake

	Means (SD)	Estimated marginal means (SE)	P value*	R^2^
**Healthy**	5.6 ± 2.5	8.1 ± 1.5	0.004	75.9
**gingivitis**	18.1 ± 6.7	15.7 ± 1.4		
*Comparison by ANCOVA; Baseline BOP, baseline 8-Hydroxy-desoxyguanosine ng/ml and plaque index after matcha tea intake were used as covariates. Significance at P value <0.05.

**Fig 4:**
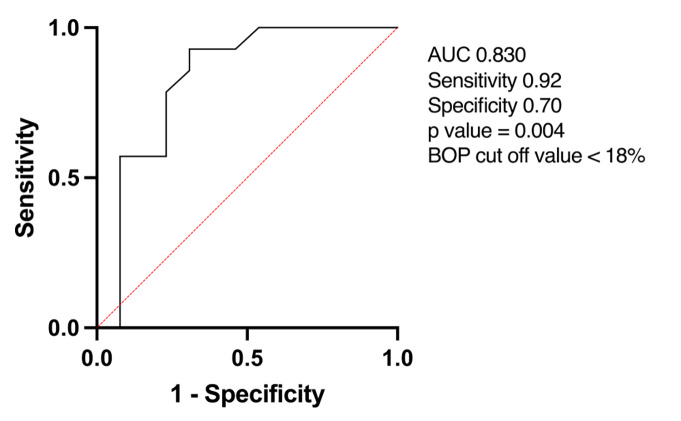
Receiver operating characteristic (ROC) analysis. Gingivitis patients with BOP <18% are predicted to have healthy gingiva (P value = 0.004; sensitivity = 0.92; specificity = 0.70) after 1 month of matcha tea intake twice a day.

In this study, it was found that patients who had localised gingivitis with a slight extent (BOP < 18%) and regularly brushed their teeth were predicted (sensitivity = 0.92 and specificity = 0.70) to have healthy gingiva after drinking matcha tea twice a day for 1 month (Fig 4).

## DISCUSSION

Recently, daily intake of matcha tea has been reported to enhance periodontal health and increase salivary antioxidants.^2^ Otherwise, in the literature, limited data are available regarding the effect of matcha tea on periodontal health. This encourages conducting this study to evaluate the impact of habitual intake of matcha tea on gingival health. Moreover, this study estimated the cut-off value of the mean BOP to predict a statistically significant reduction in mean BOP (less than 10% threshold) after 1 month of matcha tea consumption.

The main finding in this study is the statistically significant reduction of bleeding as well as PI scores in patients with localised gingivitis after regular intake of matcha tea for 1 month. This indicates the beneficial effect of matcha tea in reducing the inflammation of the gingival tissue. Many previous studies support these findings of the recent study. It is worth mentioning that matcha tea is a kind of green tea which is prepared from the same tea plant and has the same phytochemical constituents with distinct proportions.^2^ Therefore, matcha tea is supposed to have similar biological activities to those of green tea. It has been reported that both matcha and green tea intake daily enhances gingival health, helps subside gingival inflammation, and reduces bleeding.^2^ After drinking green tea twice daily, periodontitis patients were reported to have further reduction in gingival inflammation and bleeding scores after 1 month of subgingival instrumentation.^7^ Also, daily intake of green tea helps to reduce inflammation as it statistically significantly reduces salivary IL-1ß in patients with periodontitis during non-surgical periodontal therapy.^26^ On the other hand, dental biofilm is the main causative factor for the initiation and progression of gingival inflammation.^6^ In the recent study, dental biofilm was statistically significantly reduced after daily intake of matcha tea. Therefore, fewer bleeding sites were detected.

In this study, the mean PI of the participants was statistically significantly reduced after tea intake. This finding aligns with previous reports.^2,22^ The anti-plaque effect of matcha tea may be attributed to the antibacterial properties of its constituents, such as catechins. In literature, catechins have been reported to inhibit the growth of primary colonisers of dental biofilms such as *Streptococcus mitis*, *Streptococcus sobrinus* and *Actinomyces*.^13,30^ On the other hand, there is evidence supporting the retention of catechins in the oral cavity and plasma after tea intake for a prolonged period of time.^10,20,31^ Therefore, habitual daily intake of matcha tea provides a continuous source of catechins, ensuring long-standing availability of these beneficial molecules in the oral cavity. For all the above-mentioned reasons, less dental biofilm was detected on teeth due to the prolonged antibacterial effects offered by matcha tea constituents. This might retard biofilm formation on teeth and thus lower PI scores were recorded.

In inflamed tissue, 8-OHdG is produced through endogenous oxidation of DNA, contributing to its use as a biomarker to express oxidative stress in inflammatory conditions such as periodontal diseases.^14,28^ In this study, salivary 8-OHdG was found to be statistically significantly reduced after matcha tea intake. This finding is not surprising as matcha tea has been reported to have anti-oxidant effects attributed to its content of catechins, particularly (−)-epigallocatechin-3-gallate (EGCG). The daily intake of matcha tea provides a continuous source of these antioxidants, which act by neutralising oxidants in tissue and thereby balancing oxidative stress. There are several proposed mechanisms of the antioxidant effect of catechins. EGCG has a potent ability to neutralise and prevent the formation of free oxygen radicals by transferring a hydrogen atom and chelating metal ions, respectively, thus minimising their bioavailability within tissue.^3,12^ Moreover, tea polyphenols induce the production of antioxidant enzymes such as catalase and superoxide dismutase. Also, they modify the Nrf2 signalling pathway in the direction of the expression of the protective genes against oxidative damage.^21^ In conclusion, matcha tea has a protective antioxidant effect potentially attributed to its content of polyphenols, particularly catechins. This is supported by the statistically significant reduction of the tissue oxidation product 8-OHdG in this study. However, this finding was not observed in all participants. The salivary level of 8-OHdG was not statistically significantly different in individuals who still experienced gingivitis after consuming matcha tea, compared to those with healthy gingiva. This might be due to the fact that the levels of exogenous antioxidants supplied by matcha tea were not enough to neutralise a considerable amount of the oxidants in those with an extent of BOP ≥ 18%. Only patients with a slight extent of gingivitis (BOP <18%) could receive the benefits of antioxidant matcha tea for the resolution of the gingival inflammation.

This study evaluated the effect of matcha tea intake for only a relatively short period of 1 month. The participants involved in this study who had localised gingivitis were asked to drink matcha tea only, without any additional intervention. For ethical consideration, it was not possible to keep the participants without actual periodontal therapy, oral hygiene motivation and instructions, plus scaling, for a longer period. One additional limitation is that this study relied on a single clinical parameter (ie, BOP) for evaluating the status of the gingiva. It is worth noting that inaccurate recording of clinical parameters can occur in clinical settings. It was better to investigate salivary biomarkers representative of gingival inflammation, such as interleukins and matrix metalloproteinase-8, in this study. However, clinical parameters in this study were recorded by a single examiner who was calibrated to enhance the validity of the recorded data. Another limitation was the lack of a placebo group in this study. However, baseline data could partly serve this purpose as the participants had localised gingivitis and declared no history of tea intake. One more concern in this study is that the participants might have shown better performance of oral hygiene measures when they were involved in the study (Hawthorne effect). Therefore, caution should be taken when interpreting the improvement of the clinical parameters, which might be partly due to improved self-performed oral hygiene measures. Furthermore, it was better to have self-reported feedback from the participants to evaluate the impact the tea intake on gingival health after matcha tea intake. This could be suggested in future studies.

The outcomes of this study support the evidence regarding the benefits of matcha tea for promoting gingival health. In a recent clinical study, drinking matcha tea on a regular basis has been reported to reduce microbial biofilm on teeth and bleeding from the gingiva, suggesting a positive role of matcha tea in enhancing periodontal health.^2^ Moreover, rinsing the oral cavity with matcha tea has the potential to reduce periodontal pathogens in saliva.^24^ Therefore, dental practitioners may encourage patients with localised gingivitis to consume matcha tea daily, as it may potentially reduce inflammation and promote gingival health.

## CONCLUSION

Despite the limitations of this pilot study, regular matcha tea intake for 1 month has the potential to improve gingival health. Patients with localised gingivitis of a slight extent may benefit from daily matcha tea intake to help resolve gingival inflammation.

### Acknowledgement

The present study received no explicit financial support from public, commercial, or not-for-profit funding agencies.
